# The Thermoregulatory and Thermal Responses of Individuals With a Spinal Cord Injury During Exercise, Acclimation and by Using Cooling Strategies–A Systematic Review

**DOI:** 10.3389/fphys.2021.636997

**Published:** 2021-04-01

**Authors:** Fabian Grossmann, Joelle Leonie Flueck, Claudio Perret, Romain Meeusen, Bart Roelands

**Affiliations:** ^1^Human Physiology and Sports Physiotherapy Research Group, Vrije Universiteit Brussel, Brussels, Belgium; ^2^Sports Medicine, Swiss Paraplegic Centre, Nottwil, Switzerland; ^3^School of Psychology and Life Sciences, Canterbury Christ Church University, Canterbury, United Kingdom

**Keywords:** thermoregulation, thermal physiology, paralympics, acclimation, cooling, heat strain

## Abstract

**Background:** In individuals with a spinal cord injury thermoregulatory mechanisms are fully or partially interrupted. This could lead to exercise-induced hyperthermia in temperate conditions which can be even more distinct in hot conditions. Hyperthermia has been suggested to impair physiological mechanisms in athletes, which could negatively influence physical performance and subjective well-being or cause mild to severe health issues.

**Objective:** The aim was to evaluate the literature on the thermoregulatory and thermal responses of individuals with a spinal cord injury during exercise in temperate and hot conditions taking the effects of cooling techniques and heat acclimation into account.

**Data sources:** Two electronic databases, PubMed and Web of Science were searched. Studies were eligible if they observed the influence of exercise on various thermoregulatory parameters (e.g., core and skin temperature, sweat rate, thermal sensation) in individuals with a spinal cord injury.

**Results:** In total 32 articles were included of which 26 were of strong, 3 of moderate and 3 of weak quality. Individuals with a high lesion level, especially those with a tetraplegia, reached a higher core and skin temperature with a lower sweat rate. The use of cooling techniques before and during exercise can positively affect the burden of the impaired thermoregulatory system in all individuals with a spinal cord injury.

**Conclusion:** Due to the absence of normal thermoregulatory abilities, individuals with a high-level spinal cord injury need special attention when they are exercising in temperate and hot conditions to prevent them from potential heat related issues. The use of cooling techniques can reduce this risk.

## Introduction

In individuals with a spinal cord injury (SCI) the afferent input to the thermoregulatory center, i.e., hypothalamus, from below the lesion level is decreased. This inherently leads to reductions in the efferent output, causing a disruption of the cutaneous vasodilatation as well as an impaired activation of the sweat glands (List, 1938; Wallin and Stjernberg, 1984; Stjernberg et al., 1986; Hopman et al., 1993). The higher the level of the SCI, the more the afferent information regarding thermoregulation is reduced (Normell, 1974; Claus-Walker and Halstead, 1981). Thus in persons with a cervical SCI [i.e., tetraplegia (TP)] the brain receives a smaller amount of afferent information, and therefore, less efferent information is available for the periphery, compared to individuals with a thoraic, lumbar or sacral SCI [i.e. paraplegia (PA)] (Guttmann et al., 1958). Beside the decreased input to the thermoregulatory center, individuals with a TP have a lower available or recruitable muscle mass, which has a direct influence on the total heat production. This difference can also be demonstrated by physiological variables like oxygen consumption and energy expenditure (Price and Trbovich, 2018). Campagnolo et al. (2011) published excellent book which covers the key physiological adaptations following a SCI in more detail. During exercise in temperate (18–25°C) conditions, and even more in hot conditions (>25°C), athletes with a SCI are adversly affected by the lack of afferent and efferent thermoregulatory information (Price, 2006). As a consequence, the internal and external heat load might have a greater impact, resulting in a greater potential for exercise-induced hyperthermia with subsequent performance decrements, as well as the risk of potential heat illness (e.g., heat stroke). Based on current knowledge it is more likely in individuals with a high lesion level (i.e., those with TP) (Price, 2006; Price and Trbovich, 2018). Therefore, besides the use of monitoring techniques, cooling strategies have been applied to decrease the risk for such hyperthermic symptoms and to induce optimization of performance regarding a less pronounced increase in deep-body temperature (Tc). Also, acclimation or acclimatization might be of interest, even though scientific evidence from able-bodied (AB) athletes cannot be adopted unaltered to athletes with a SCI (Gorgey et al., 2014).

The main reason for systematically reviewing the literature was to gather information on thermoregulatory and thermal responses of individuals with PA, TP on exercise-induced heat stress while exercising in temperate and hot conditions. Since 2006 (Price, 2006) it is the first time literature on this topic was reviewed systematically. Although the cooling techniques were recently reviewed by Griggs et al. (2015b), as secondary aim we reviewed evidence for the thermoregulatory and thermal responses during the use of cooling strategies as well as during heat acclimation. To date no wide-ranging systematic review on the thermoregulatory responses of individuals with SCI during exercise in different conditions is available. Therefore, the outcome of the current work (i.e., on adequate cooling strategies) should help athletes and their coaches in the preparation for a successful participation at the upcoming Paralympic Games in Tokyo or other major championships in the heat and provides indications for researchers as well.

## Methods

### Eligibility Criteria

PICOS (Table 1) critera were used to include studies performed as randomized controlled trials (RCTs), non-randomized controlled trials (nRCTs) and non-randomized non-controlled trials (nRnCTs) (Moher et al., 2015). These studies had to investigate thermoregulatory responses of individuals with a SCI during exercise. Studies without any exercise task, without collecting data of Tc or animal studies were excluded. Only original studies written in English language were included.

**Table 1:** PICOS (participants, interventions/exposure, comparison, outcomes, study design).

**PICOS components**	**Detail**
**P**articipants	Humans with a spinal cord injury able (classification-wise) to participate in Paralympic wheelchair sports
**I**nterventions/Exposure	Exercise in normal or hot conditions
**C**omparison	Able-bodied vs. para-/tetraplegic, paraplegic vs. tetraplegic, cooling vs. non-cooling, acclimation/acclimatization vs. non-acclimation, descriptive
**O**utcomes	Performance, thermoregulatory responses
**S**tudy designs	RTCs, nRCTs and nRnCTs

*RTCs, randomized controlled trials; nRCTs, non-randomized controlled trials; nRnCTs, non-randomized non-controlled trials*.

### Information Source and Search Strategies

Two online databases, Web of Science and PubMed (until 30.09.2020), were searched. The following keywords were applied individually and combined: Paralympic, spinal cord injured, spinal cord injury, paraplegic, tetraplegic, quadriplegic, paraplegia, tetraplegia, quadriplegia, wheelchair, hand-bike, thermoregulation, body temperature, hyperthermia, exercise, sport and physical activity (Table 2). Additionally, the listed references of the included studies were screened.

**Table 2:** Number of hits on keywords and combined keywords in PubMed and Web of Science.

**Keywords**	**PubMed**		**Web of Science**	
	**Hits (30.09.20)**	**Selected articles**	**Hits (30.09.20)**	**Selected articles**
(1) Paralympic OR spinal cord injured OR spinal cord injury OR paraplegic OR tetraplegic OR quadriplegic OR paraplegia OR tetraplegia OR quadriplegia OR wheelchair OR handbike	105′490	NA	145′990	NA
(2) thermoregulation OR thermoregulatory OR body temperature OR hyperthermia	367′256	NA	525′411	NA
(3) Exercise OR physical activity OR sport	753′472	NA	1′570′806	NA
Combined keywords				
(1) AND (2)	561	NA	1′305	NA
(1) AND (3)	2′173	NA	12′894	NA
(1) AND (2) AND (3)	160	22	264	10

### Study Selection and Data Collection Process

**Figure 1:**
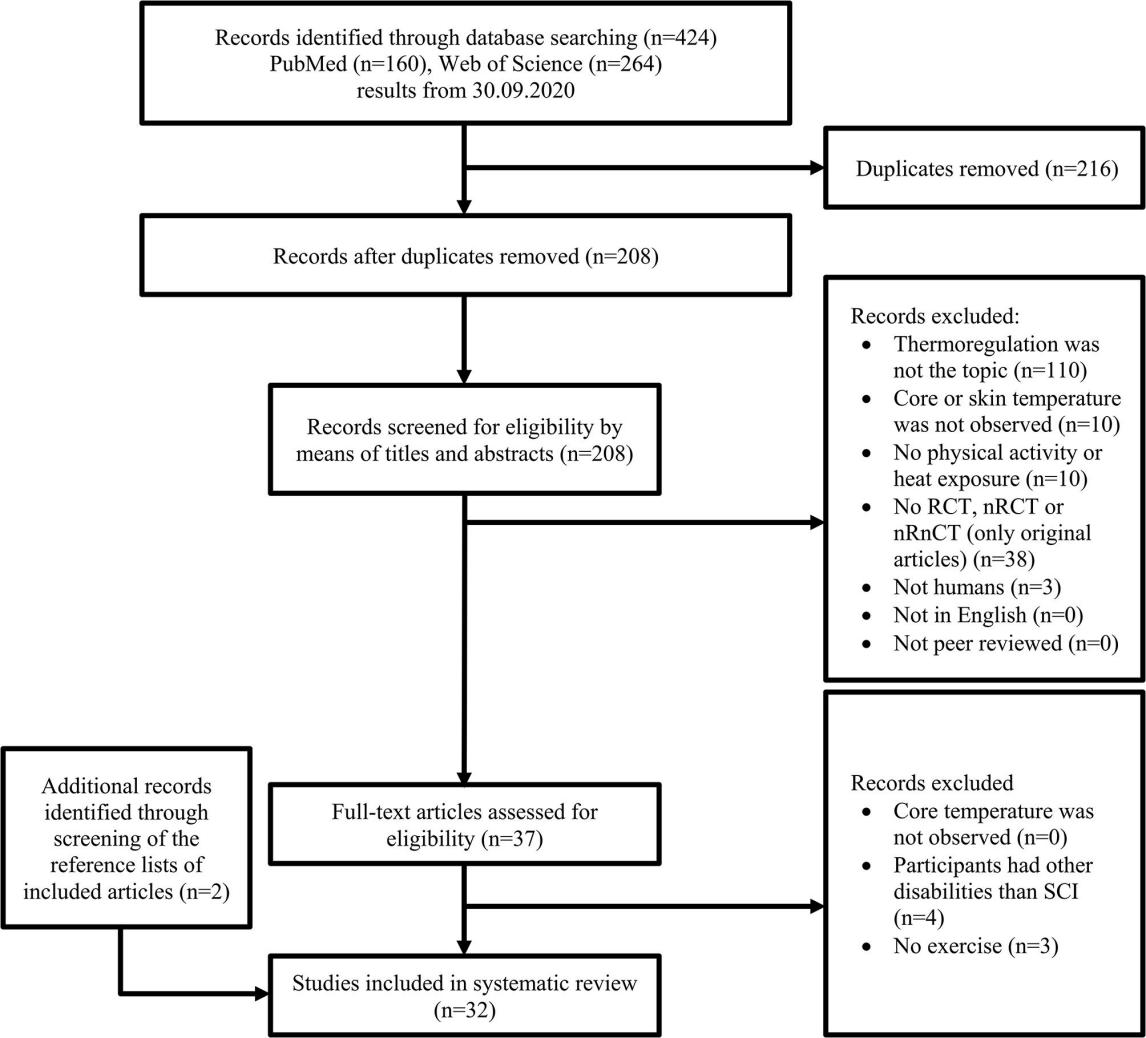
Selection process for research articles (*n* = 32) included in this systematic review. This version is adapted from the recommendation in the PRISMA (Preferred Reporting Items for Systematic Reviews and Meta-Analyses) statement. RCT, randomized controlled trial; nRCT, non-randomized controlled trial; nRnCT, non-randomized non-controlled trial; SCI, spinal cord injury.

After removing all duplicates, records were screened for eligibility including title, abstract and/or full text. The titles and abstracts were screened first, followed by considering the full texts for potentially relevant articles. The data collection process is presented in Figure 1 (Moher et al., 2015).

### Quality Assessment

Methodological quality assessment was conducted using the quantitative assessment tool “QualSyst” by Kmet et al. (2004). Studies were scored by means of 14 items. Each item was rated with yes = 2, partial = 1 or no = 0, depending on its specification. Criteria not applicable to a particular study design were marked with “N/A” and were excluded from the calculation of the summary score. For each study a summary score was computed by summing up the total score across the relevant items and dividing it by the total possible score. Three reviewers (FG, JF, CP) independently performed this quality assessment. In order to find a consensus in case of disagreements, articles were extensively discussed by the three reviewers. A score of > 75% indicated strong quality, 55–75% moderate quality and <55% weak quality. Quality scores were presented in a comprehensive table (Table 3).

**Table 3:** Quality assessment “QualSyst” (Kmet et al., 2004).

**Study**	**Question described**	**Appropriate study design**	**Appropriate participant selection**	**Characteristics described**	**Random allocation**	**Researchers blinded**	**Participants blinded**	**Outcome measures well defined and robust to bias**	**Sample size appropriate**	**Analytic methods well described**	**Estimate of variance reported**	**Controlled for confounding**	**Results reported in detail**	**Conclusion supported by results**	**Rating**
Armstrong et al. (1995)	2	2	1	1	1	NA	NA	2	0	0	2	1	2	2	Moderate
Au et al. (2019)	2	2	1	2	NA	NA	NA	2	2	2	2	1	2	2	Strong
Bongers et al. (2016)	2	2	2	2	2	0	NA	2	1	2	2	2	2	2	Strong
Boot et al. (2006)	2	2	1	2	1	NA	NA	2	1	1	2	1	2	2	Strong
Castle et al. (2013)	1	1	1	1	NA	NA	NA	2	1	1	2	0	1	1	Weak
Dawson et al. (1994)	1	1	1	2	0	NA	NA	2	1	1	2	2	2	2	Moderate
Fitzgerald et al. (1990)	2	1	1	2	NA	NA	NA	1	1	1	1	1	2	2	Moderate
Forsyth et al. (2019)	2	2	2	2	1	1	NA	2	1	2	2	1	2	2	Strong
Gass et al. (1988)	2	2	1	2	NA	NA	NA	2	1	1	2	2	2	2	Strong
Hopman et al. (1993)	2	2	1	2	NA	NA	NA	2	1	1	2	1	2	2	Strong
Griggs et al. (2015a)	2	2	1	2	NA	NA	NA	2	2	2	2	1	2	2	Strong
Griggs et al. (2017a)	2	2	2	2	0	NA	NA	2	1	2	2	1	2	2	Strong
Griggs et al. (2017b)	2	2	2	2	NA	NA	NA	2	2	2	2	1	2	2	Strong
Hagobian et al. (2004)	2	2	1	2	1	NA	0	2	1	1	2	1	2	2	Strong
Iturricastillo et al. (2018)	2	2	1	2	NA	NA	NA	2	1	1	2	1	2	2	Strong
Petrofsky (1992)	1	0	1	0	1	NA	NA	2	1	1	0	1	1	1	Weak
Price and Campbell (1997)	2	2	1	1	NA	NA	NA	2	2	1	2	1	2	2	Strong
Price and Campbell (1999a)	2	2	1	2	2	NA	NA	2	1	1	2	2	2	2	Strong
Price and Campbell (1999b)	2	2	1	2	NA	NA	NA	2	1	1	2	2	2	2	Strong
Price and Campbell (2003)	2	2	2	1	NA	NA	NA	2	2	1	2	2	2	2	Strong
Pritchett et al. (2010)	2	2	1	2	1	NA	NA	2	1	1	2	2	2	2	Strong
Pritchett et al. (2011)	2	2	2	2	NA	NA	NA	2	2	1	2	2	2	2	Strong
Pritchett et al. (2015)	2	2	1	2	NA	NA	NA	2	1	1	2	2	2	2	Strong
Theisen et al. (2000)	2	2	2	2	NA	NA	NA	2	1	1	2	2	2	2	Strong
Theisen et al. (2001)	2	2	1	2	NA	NA	NA	2	1	1	2	2	2	1	Strong
Trbovich (2019)	2	1	1	0	1	NA	NA	2	0	1	NA	1	2	1	Weak
Trbovich et al. (2014)	2	1	1	2	2	NA	NA	2	1	1	2	2	2	2	Strong
Trbovich et al. (2016)	2	1	2	2	NA	NA	NA	2	1	2	2	1	2	2	Strong
Trbovich et al. (2019)	2	2	2	2	NA	NA	NA	1	1	2	2	1	2	1	Strong
Veltmeijer et al. (2014)	2	2	1	2	NA	NA	NA	2	1	1	2	1	2	2	Strong
Webborn et al. (2005)	2	2	1	2	2	NA	NA	2	1	1	2	2	2	2	Strong
Webborn et al. (2010)	2	2	1	2	2	NA	NA	2	1	2	2	2	2	2	Strong

*NA not applicable, 2 indicates yes, 1 indicates partial, 0 indicates no*.

*Quality scores: > 75% strong, 55% ≥ 75% moderate, <55% weak*.

## Results

### Study Selection

The search strategy resulted in a total of 424 hits. After removing duplicates as well as examining titles and abstracts for inclusion and exclusion criteria, 37 articles remained for inclusion. Screening of their full texts led to seven exclusions. By screening the reference list of the remaining 30 articles two additional articles were included (Figure 1). The quality assessment of the 32 selected articles led to 26 articles of strong, three of moderate and three of weak quality (Table 3). In the following sections (3.2–3.4) and in the discussion the included studies were split up into four areas: Thermoregulatory and thermal responses in temperate conditions, thermoregulatory and thermal responses in hot conditions, thermoregulatory and thermal responses by applying cooling methods and heat acclimation. No studies were included in more than one category.

### Thermoregulatory and Thermal Responses in Temperate Conditions

In total 14 articles (Table 4) were included investigating the effects of exercise during temperate conditions (range: 18.4 to 25.0°C and 31 to 65% relative humidity) on the thermoregulatory and thermal response in individuals with SCI. The exercise type was different between the studies and ranged from arm cranking (Price and Campbell, 1997, 1999a; Theisen et al., 2000, 2001; Pritchett et al., 2011, 2015; Veltmeijer et al., 2014; Griggs et al., 2017a,b), wheelchair ergometry (Fitzgerald et al., 1990; Price and Campbell, 1999a), wheelchair on treadmill (Gass et al., 1988) to simulated or real games (Veltmeijer et al., 2014; Iturricastillo et al., 2016; Griggs et al., 2017b). The protocols varied between continuous and intermittent bouts, while the total duration of the exercise load ranged between 16 and 90 min. All but three studies included AB persons as a control or comparison group (Gass et al., 1988; Price and Campbell, 1999a; Griggs et al., 2015a). Individuals studied were heterogeneous regarding fitness and lesion level. Measurement of body Tc included methods such as intestinal (Veltmeijer et al., 2014; Griggs et al., 2015a, 2017b; Au et al., 2019), oral (Fitzgerald et al., 1990), esophageal (Gass et al., 1988; Theisen et al., 2000, 2001; Pritchett et al., 2010, 2011; Au et al., 2019), rectal (Gass et al., 1988; Pritchett et al., 2010, 2011), tympanic (Iturricastillo et al., 2016) and aural (Price and Campbell, 1997, 1999a,b; Griggs et al., 2017a) temperature measurements.

**Table 4:** Thermoregulatory responses in thermal neutral conditions.

**Study**	**Sample**	**Climate**	**Intervention/Exposure**	**Duration**	**Thermal measurement**	**Methodological characteristics**	**Outcome**
**Part 1**							
Au et al. (2019)	5 M, PA (T4-T11) 7 M, TP (C4-C7) 5 M, AB	25°C 50% rh	30′ arm cranking exercise @ 50% VO_2_peak	10′ rest 2x 15′ 2′ rest between bouts	T_eso_ T_int_ T_sk_	nRnCT cohort study	T_eso_/T_int_ in TP → , PA ↑ T_eso_/T_int_ no Δ between PA and AB T_sk_ upper limb → T_sk_ lower limb →
Fitzgerald et al. (1990)	5 F, PA 5 F, AB	24–25°C 38–52% rh	90′ WC ergometry 50–55% VO_2_peak	30′ rest 90′ exercice	T_or_ T_sk_	nRnCT cohort study	Δ T_or_ ↑ in PA Δ T_sk_ ↑ in PA
Gass et al. (1988)	6 M, PA (T10-T12)	22°C	45′ WC treadmill with at 60-65% VO_2_max difference between T_eso_ and T_re_	10′ rest 45′ exercise 10′ rest	T_eso_ T_re_	nRnCT cohort study	Faster rise and higher T_eso_ vs. T_re_
Griggs et al. (2015a)	8 TP (C4-C7) 8 PA (T4-S1)	20.6 ± 0.1°C 39.6 ± 0.8% rh	55.5′ ISP 4 exercise blocks à 6 times (15′′ forward/ backward pushing, 15′′ sprint, 90′′ active recovery) 4.5′ in between passive recovery	10′ WU55.5′ ISP15′ rest	T_c_ T_sk_ ThS Fluid loss Performance	nRnCT cohort study	T_c_ ↑ in TP than PA ΔT_c_ ↑ in TP than PA T_c_ remained elevated in TP at rest ΔT_sk_ ↑ in TP; TP ↑, PA ↓ ThS → Fluid loss → Performance →
Griggs et al. (2017b)	10 TP (C5-C7) 7 Non-SCI	18.4–20.9°C 31.1–45.1% rh	WC rugby game	4x 8′ effective	T_c_ T_sk_ ThS Fluid loss	nRnCT cohort study	ΔT_c_ ↑ TP ΔT_sk_ ↑ TP ThS → Fluid loss ↑ TP
Iturricastillo et al. (2016)	6 SCI (C5-L3) 6 Non-SCI	NA (in sports hall)	16′ small sided games	4x 4′ separated by 2′ rest	T_y_	nRnCT cohort study	T_y_→
Price and Campbell (1999a)	7 PA (T3-L1)	21.5 ± 1.3°C 54.2 ± 6.3% rh	60′ arm cranking and WC ergometer at 60% VO_2_peak	15′ rest 60′ exercise 30′ rest	T_au_ T_sk_ Fluid loss	RCT cross-over	ΔT_au_ → between arm cranking and wheelchair ergometer T_sk_ upper arm ↑ in arm cranking Fluid loss →
Price and Campbell (1999b)	9 PA (T3-L1) 1 TP (C6) 11 AB	21.5 ± 1.7°C 49.0 ± 6.3% rh	60 ′ arm cranking at 60% VO_2_peak	15′ rest 5′ WU 60′ exercise 30′ recovery	T_au_ T_sk_ Fluid loss	nRCT cohort study	ΔT_au_ → AB and PA ΔT_au_ ↑ in TP T_au_ ↑ during recovery in TP ΔT_sk_ upper body → in all groups ΔT_sk_ tight ↑ in AB T_sk_ calf ↓ in AB Fluid loss ↓ in TP
Price and Campbell (1997)	10 PA (T3-L4) 9 AB	21.5 ± 1.7°C 47 ± 7.8% rh	90′ arm cranking at 80% peak HR	15′ rest 5′ WU 90′ exercise 20′ rest	T_au_ T_sk_ Fluid loss	nRCT cohort study	ΔT_au_ → AB and PA T_sk_ upper body ↑ PA, ↓ AB T_sk_ calf ↑PA ↓AB Fluid loss →
Pritchett et al. (2015)	7 PA 8 AB	21 ± 1°C 55-65 ± 0.1% rh	Incremental stage test 7′/stage; start 35 W, increment 35 W, till T_es_ rose more 0.2°C/min or 90 W	7′ each stage 1′ in-between passive recovery Mean test duration 27′ 30′′	T_eso_ T_re_ Sweat gland density Sweat rate	nRnCT cohort study	Active sweat gland density ↑ in AB vs. PA Sweat rate per gland → T_eso_ → T_re_→
Pritchett et al. (2011)	7 PA 8 AB	20 ± 1°C 45-65 ± 0.1% rh	Incremental stage test 7′/stage; start 35 W, increment 35 W, till T_es_ rose more 0.2°C/min or 90 W	7′ each stage 1′ in-between passive recovery	T_eso_ T_re_ T_sk_	nRnCT cohort study	ΔT_eso_ → AB and PA ΔT_re_ → AB and PA T_sk_ ↑ PA
Theisen et al. (2000)	6 M, PA, (T5-T9) 6 M, PA, (T10-T12), 6 AB	22.6 ± 1.7°C 45-65 ± 2.9% rh	Incremental stage test: 3′/stage; start 15 W, increment 20 W, till exhaustion	30‘rest 3‘stages	T_eso_ T_sk_	nRCT	ΔT_eso_ ↑ PA, at higher intensities only in PA (T10-T12) T_sk_ leg → in PA T_sk_ leg ↓ in AB

*M, male; F, female; AB, able-bodied; PA, paraplegia; TP, tetraplegia; SCI, spinal cord injured; C, cervical; T, thoracic; L, lumbar; WC, wheelchair; °C, degree Celsius; rh, relative humidity; WU, warm-up; T_re_, rectal temperature; T_eso_, esophageal temperature; T_sk_, skin temperature; T_int_, intestinal temperature; T_y_, tympanic temperature; T_au_, aural temperature; ThS, thermal sensation; RCT, randomized controlled trial; nRnCT, non-randomized non-controlled trial; W, watt; P_max_, maximal power output; HR, heart rate; ↑ = higher; → = indifferent; ↓ = lower; Δ = difference; ISP, intermittent sprint protocol*.

Three (Price and Campbell, 1999b; Griggs et al., 2015a, 2017b) out of four articles including individuals with TP, detected a larger rise in Tc for TP compared to AB participants (i.e., mean increase Tc of 1.6°C in TP vs. 0.7°C in AB) and counterparts with PA (range: 0.9–1.2°C vs. 0.5–0.7°C in TP vs. PA). Three articles observed a bigger increase in Tc in individuals with a PA (range 0.4–0.9°C) compared to AB (range: 0.0–0.5°C) controls (Fitzgerald et al., 1990; Theisen et al., 2000, 2001). In contrast, five articles did not report any differences between AB and PA individuals (Price and Campbell, 1997; Pritchett et al., 2011, 2015; Veltmeijer et al., 2014; Iturricastillo et al., 2016). Two out of 14 (Gass et al., 1988; Iturricastillo et al., 2016) studies did not monitor mean or site skin temperature (Tsk). In insensate body parts, TP and PA reached significantly higher Tsk compared to the sensate areas or AB. Sweat rate or fluid loss was investigated by six studies (Price and Campbell, 1997, 1999a,b; Griggs et al., 2015a, 2017b; Pritchett et al., 2015). The studies showed a lower sweat rate in TP compared to AB and PA. Thermal sensation (ThS) was reported in three out of 14 articles (Veltmeijer et al., 2014; Griggs et al., 2015a, 2017b) without detecting any difference between the groups.

### Thermoregulatory and Thermal Responses in Hot Conditions

Six studies (Table 5) investigated the thermoregulatory and thermal response during an exposure to hot conditions (temperature ranged between 30 and 40°C with a relative humidity of 33 to 70%). The chosen exercise type varied between arm cranking (Petrofsky, 1992; Dawson et al., 1994; Boot et al., 2006; Forsyth et al., 2019), wheelchair ergometry (Price and Campbell, 2003) and wheelchair exercise on a treadmill (Gass et al., 2002). All studies selected a continuous protocol, while the duration ranged between 30 and 60 min. Exercise intensity was determined by VO_2_ (Dawson et al., 1994; Price and Campbell, 2003), power output (Petrofsky, 1992; Gass et al., 2002; Boot et al., 2006) or fixed heat production (Forsyth et al., 2019). All studies considered a rest period prior to the exercise and entering the climatic chamber. This time period varied between 5 and 30 min. Two studies compared TP with PA and AB individuals (Petrofsky, 1992; Forsyth et al., 2019), one compared TP with PA participants (Price and Campbell, 2003), two compared PA with AB persons (Dawson et al., 1994; Boot et al., 2006) and one observed only PA participants (Gass et al., 2002). Different levels of PA (e.g., high lesion level and low lesion level PA) were compared by Price and Campbell (2003); Boot et al. (2006) and Forsyth et al. (2019). Rectal (Dawson et al., 1994; Gass and Gass, 2001; Boot et al., 2006), esophageal (Forsyth et al., 2019) or aural methods (Petrofsky, 1992; Price and Campbell, 2003) were used to measure Tc. The change in Tc was greater (range: 1.2–2.1°C vs. 0.9–1.2°C vs. 0.3°C in TP, PA and AB) for TP. Additionally, from similar baseline values TP reached greater Tc compared to PA and AB participants (40.3°C vs. 38.9°C vs. 38.0°C in TP, PA and AB) (Petrofsky, 1992; Price and Campbell, 2003; Forsyth et al., 2019). Only one study failed to detect any differences in measured Tc values between PA and AB individuals (Dawson et al., 1994). All but one (Petrofsky, 1992) investigation recorded Tsk and showed to be greater in body parts where the sweat response is impaired. All studies included the measurement of fluid loss and reported lower sweat rates in PA compared to AB and TP compared to PA. No study recorded thermal sensation.

**Table 5:** Thermoregulatory responses in hot conditions.

**Study**	**Sample**	**Climate**	**Intervention/Exposure**	**Duration**	**Thermal measurement**	**Methodological characteristics**	**Outcome**
Boot et al. (2006)	4 M, PA, (T1-T6) 6 M, PA, (T7-T12) 10 M, AB	10°C 65% rh 35°C 70%	45′ arm cranking @ 40% P_peak_ in two different climates	20′ sitting in climate chamber 45′ exercise	T_re_ T_sk_ Fluid loss	nRnCT cohort study	ΔT_re_ ↑ PA in 35° ΔT_sk_ lower body ↑ in PA (T1-T6) in 35° ΔT_sk_ no differences Fluid loss ↑ in AB
Dawson et al. (1994)	5 M, PA (T12-L1) 5 M, AB	37.4 ± 0.3°C 33.0 ± 1.9% rh 15.0 ± 0.2°C 56.9 ± 2.1% rh	60′ arm cranking @ 55-60% VO_2_max	5′ in climate chamber 60′ exercise	T_re_ T_sk_ Fluid loss	nRnCT cohort study	ΔT_re_ no differences in both conditions ΔT_sk_ mean ↑ in AB in heat Fluid loss → ; slightly higher in AB
Forsyth et al. (2019)	9 TP 7 PA, (T1-T5) 8 PA, (T6-L1) 8 AB	35°C 50% rh	30′ arm cranking @ fixed heat production 4.0 W/kg TP/AB 6.0 W/kg PA/AB	30′ rest (25°) 30′ exercise 3-min rest every 10^th^ minute	T_eso_ T_sk_ sweat rate	nRnCT cohort study	ΔT_eso_ ↑ in TP than AB ΔT_eso_ ↑ in PA than AB ΔT_sk_ chest/shoulder ↑ in TP than AB ΔT_sk_ lower body ↑ in TP than AB ΔT_sk_ calf ↑ in PA than AB sweat rate → in AB and PA no sweat rate in TP
Hopman et al. (1993)	13 PA (T2-T12)	35°C ± 0.5°C 70 ± 5% rh	45′ arm cranking @ 40% P_peak_	45′ exercise	T_re_ Fluid loss	nRnCT cohort study	ΔT_re_ → Fluid loss ↑ in PA
Petrofsky (1992)	6 M, PA (T3-T12) 6 M, TP (C6-C8) 4 AB	30/35/40°C	30′ arm cranking @ 50 W or cycle ergometry under computer control @ 50 W	30′ rest 30′ arm cranking or cycling	T_au_ Sweat rate	RCT cross-over	↑ T_au_ in SCI than AB for both exercise in 35 and 40°C ↑ Sweat rate in AB than SCI for both exercises in all conditions
Price and Campbell (2003)	8 TP (C5-C8) 10 PA, (T1-T6) 10 PA, (T7+below)	31.5 ± 1.7°C 42.9 ± 8.0% rh	60′ WC ergometry @ 60% VO_2_peak	15′ rest in normal condition 10′ rest in hot condition 5′ WU 60′ exercise in heat 30′ rest	T_au_ T_sk_ Fluid loss	nRnCT cohort study	T_au_ ↑ in TP end of exercise T_au_ no difference between the two PA groups T_au_ remained elevated T_sk_ back ↑ in TP T_sk_ → between the two PA groups Fluid loss ↑

*M, male; AB, able-bodied; PA, paraplegia; TP, tetraplegia; SCI, spinal cord injury; C, cervical; T, thoracic; L, lumbar; WC, wheelchair; rh, relative humidity; °C, degree Celsius; WU, warm-up; T_re_, rectal temperature; T_eso_, esophageal temperature; T_sk_, skin temperature; T_int_, intestinal temperature; T_y_, tympanic temperature; T_au_, aural temperature; RCT, randomized controlled trial; nRnCT, non-randomized non-controlled trial; W, watt; HR, heart rate; P_peak_, peak power output; ↑ = higher; → = indifferent; ↓ = lower*.

### Thermoregulatory and Thermal Responses With Cooling

Ten articles focused on the effect of cooling during (DUR) or cooling before an activity (PRE) (Table 6). While three studies investigated DUR and PRE (Webborn et al., 2005, 2010; Griggs et al., 2017a), six studies examined only DUR (Armstrong et al., 1995; Hagobian et al., 2004; Pritchett et al., 2010; Trbovich et al., 2014; Bongers et al., 2016; Trbovich, 2019). Environmental conditions during the intervention varied between 20.2°C and 32.9°C with a relative humidity between 26 and 75%. The chosen exercise types ranged from arm cranking (Hagobian et al., 2004; Webborn et al., 2005, 2010; Pritchett et al., 2010; Bongers et al., 2016), wheelchair exercise on a stationary roller (Armstrong et al., 1995) or in a sports hall (Griggs et al., 2017a) and on a treadmill (Trbovich, 2019) to a simulated game (Trbovich et al., 2014). The selected protocols were continuous or intermittent, whereas their duration ranged between 28 and 90 min. The effectiveness of cooling vests (Armstrong et al., 1995; Webborn et al., 2005, 2010; Trbovich et al., 2014; Bongers et al., 2016; Griggs et al., 2017a; Trbovich, 2019), a refrigerated headpiece (Armstrong et al., 1995), water spray (Pritchett et al., 2010; Griggs et al., 2017a; Trbovich, 2019), foot cooling (Hagobian et al., 2004), and ice slurry ingestion (Trbovich, 2019) have been investigated. Each study had a non-cooling crossover control trial or group. Participant groups were heterogeneous in terms of age, lesion level or training level. Two articles included only PA (Pritchett et al., 2010; Bongers et al., 2016), three studies included only TP (Webborn et al., 2005, 2010; Griggs et al., 2017a), one study compared PA with AB controls (Hagobian et al., 2004) and three studies compared TP with PA and AB, respectively (Trbovich et al., 2014, 2019; Trbovich, 2019). Rectal (Armstrong et al., 1995; Pritchett et al., 2010), aural (Armstrong et al., 1995), intestinal (Webborn et al., 2005, 2010; Trbovich et al., 2014; Bongers et al., 2016; Griggs et al., 2017a; Trbovich, 2019), tympanic (Hagobian et al., 2004) or esophageal (Pritchett et al., 2010; Cruz and Blauwet, 2018) temperature measurements were used to analyze Tc. Each article except two (Trbovich et al., 2014; Trbovich, 2019) has observed Tsk. Fluid loss were recorded in three articles (Armstrong et al., 1995; Bongers et al., 2016; Griggs et al., 2017a). Subjective perception (ThS) was recorded in five studies (Armstrong et al., 1995; Webborn et al., 2005; Pritchett et al., 2010; Bongers et al., 2016; Griggs et al., 2017a).

**Table 6:** Thermoregulatory responses using cooling techniques.

**Study**	**Sample**	**Climate**	**Intervention/Exposure**	**Duration**	**Thermal measurement**	**Methodological characteristics**	**Outcome**
Armstrong et al. (1995)	6 PA (C6-T12)	32.9 ± 0.1°C 75 ± 3% rh	30′ WC on stationary roller @ self-selected 10 km pace (67-70% VO_2_ peak) DUR with headpiece or DUR with IV	20-25′ rest in heat 30′ exercise	T_re_ T_au_ T_sk_ Sweat rate ThS	RCT cross-over	No differences between tests in T_re_, T_au_, T_sk_, sweat rate, ThS
Bongers et al. (2016)	10 M, PA (T4-T12)	25.4 ± 0.4°C 41.0 ± 8.4% rh	45′ arm cranking @ 50% peak power with DUR with IV	10′ rest in heat 10′ WU 45′ test 5′ cool down 10′ rest in heat	T_in_ T_sk_ Fluid loss ThS	RCT cross-over	T_in_ no between test differences T_sk_ mean ↓ with IV ThS ↓ with IV Fluid loss: no differences between tests
Griggs et al. (2017a)	1 F, TP 7 M, TP (C5-C7)	20.2 ± 0.2°C 33.0 ± 3.1% rh	4x 15′ intermittent sprint protocol 4x 15′ with different sprint bouts in 3 conditions No cooling PRE with IV (incl. during WU) PRE with IV and DUR with WS	15′ rest 20′ WU 4x 15′ exercise with 2′/5′/2′ rest in between	T_in_ T_sk_ ThS Fluid loss	RCT cross-over	ΔT_in_ ↓ in PRE/DUR condition T_sk_ mean ↓ in PRE/DUR condition No differences between tests in ThS,fluid loss
Hagobian et al. (2004)	6 M, SCI (C5-T5) 6 M, AB	31.8 ± 0.2°C 26 ± 1% rh	45′ arm cranking @ 66 ± 5% VO_2_peak DUR with foot cooling	30′ rest in heat 10′ rest in heat (baseline) 45′ exercise 30′ passive recovery	T_y_ T_sk_	RCT cross- over	ΔTy ↑ in non-cooling ΔTy ↑ in cooling compared to AB T_sk_ face ↑ in SCI T_sk_ back ↑ in SCI
Pritchett et al. (2010)	3 M, PA (T3-L1) 4 F, PA (T3-L1)	22 ± 1°C 45-50 ± 0.1% rh	Incremental stage test 7′/stage; start 30 W, increment 20 W, till T_es_ rose more 0.2°C/min or 90 W DUR with WS	7′ each stage 1′ in-between passive recovery	T_eso_ T_re_ T_sk_ ThS	RCT cross-over	No differences between tests in in T_re_, T_sk_, ThS, T_eso_
Trbovich (2019)	1 TP 1 PA 1 AB	75°F control 90°F hot	30′ WC treadmill 5 different conditions: control, hot, hot with IV, hot with WS, hot with ice slurry	10′ rest 30′ treadmill	T_in_	RCT cross-over	All three cooling interventions mitigated the rise in T_in_ in TP WS and ice slurry mitigated the rise in PA
Trbovich et al. (2014)	6 TP (C5-C7) 5 PA, (T3-T5) 6 PA, (T7-T12) 19 AB	21.1–23.9°C	Playing WC basketball or rugby DUR with IV Without cooling	20′ rest 60′ exercise	T_in_	RCT cross-over	T_in_ ↑ in TP than PA, AB No between test differences in T_in_ for any group or condition
Trbovich et al. (2019)	7 TP (C5-C7) 4 PA (T4–L1) 16 AB	19-22°C 55-60% rh	90′ intermittent sprint exercise under two conditions DUR with WS application every 15′ without cooling TP played WC rugby PP played WC basketball	15′ WU 60-90′ WC basketball or rugby	T_in_	RCT cross-over	ΔT_in_ ↑ in TP than PA, AB ΔT_in_ ↓ in TP with WS
Webborn et al. (2005)	8 M, TP (C5-C7)	32.0 ± 0.1°C 50.0 ± 0.1% rh	14x 2′ intermittent sprint protocol arm cranking PRE with IV DUR with IV	15′ rest in temperate 20′ IV or rest in thermal neutral 7′ WU 14x 2′ exercise	T_in_ T_sk_ ThS	RCT cross-over	T_in_ ↑ without cooling ΔT_in_ ↓ lower in DUR than other conditions T_sk_ ↓ with DUR than control ThS no differences between tests
Webborn et al. (2010)	8 M, TP (C5-C7)	32.0 ± 0.1°C 50.0 ± 0.1% rh	30x 2′ intermittent sprint protocol arm cranking PRE with IV DUR with IV	15′ rest in thermal neutral 20′ IV or rest in thermal neutral 7′ WU Up to 30x 2′ exercise	T_c_ T_sk_ Performance	RCT cross-over	T_in_ ↑ without cooling T_sk_ ↓ with DUR than control Performance ↑ with PRE than control Performance ↑ with DUR than PRE

*M, male; F, female; AB, able-bodied; PA, paraplegic; TP, tetraplegic; SCI, spinal cord injury; C, cervical; T, thoracic; L, lumbar; WC, wheelchair; °C, degree Celsius; °F, degree Fahrenheit; rh, relative humidity; WU, warm-up; T_re_, rectal temperature; T_eso_, esophageal temperature; T_sk_, skin temperature; T_int_, intestinal temperature; T_y_, tympanic temperature; T_au_, aural temperature; ThS, thermal sensation; HR, heart rate; RCT, randomized controlled trial; nRnCT, non-randomized non-controlled trial; W, watt; P_max_ = maximal power output; ↑ = higher; → = indifferent; ↓ = lower; DUR = cooling during exercise; PRE = cooling before exercise; IV = ice vest/cooling vest; WS = water spray*.

#### Cooling Vests

The use of cooling vests as a PRE device was applied in three studies. Griggs et al. (2017a) and Webborn et al. (2005, 2010) could not detect any effect on baseline Tc, whereas all three found a significantly lower increase in Tc during exercise (0.8–1.7°C in cooling vs. 1.2–1.9°C in non-cooling) after PRE. Additionally, Griggs et al. (2017a) and Webborn et al. (2005, 2010) found that mean Tsk was significantly lower after PRE with an ice vest (1.3–1.7°C). In contrast to the difference in Tsk between cooling and non-cooling period, no difference was found in Tsk after warm up (Griggs et al., 2017a). Moreover, Webborn et al. (2005, 2010) found no differences concerning mean Tsk during the exercise task. None of these three studies detected any significant differences regarding ThS over the complete exercise duration.

Using an ice or an evaporative cooling vest during exercise did not show a significant effect on Tc in the studies of Armstrong et al. (1995); Bongers et al. (2016) and Trbovich et al. (2014) over the whole exercise duration. Conversely, works of Trbovich (2019) as well as of Webborn et al. (2005, 2010) resulted in a significantly lower Tc for TP participants after using an ice vest DUR (37.0°C vs. 37.3°C in cooling and non-cooling) (Webborn et al., 2005, 2010).

Bongers et al. (2016) and Webborn et al. (2005, 2010) detected significant differences in Tsk between cooling during exercise and control. In contrast, Armstrong et al. (1995) could not find any differences. Bongers et al. (2016) reported a trend for a lower ThS comparing cooling with an ice vest and non-cooling during exercise.

#### Head Cooling

Only one study investigated the effect of wearing a refrigerated head piece during exercise (Armstrong et al., 1995). The authors could not find any significant variation in Tc and Tsk between cooling and non-cooling. No difference in ThS was observed.

#### Foot Cooling

The only study that examined the effect of using a foot cooling device during arm cranking, detected a significantly lower increase of Tc (1.6°C non-cooling, 1.0°C cooling) during exercise compared with non-cooling. In AB no difference was detected (Hagobian et al., 2004). In regard of Tsk, there was no significant difference.

#### Water Spray

The use of water spray as artificial sweat during exercise was examined in two studies. In one study, Trbovich (2019) found the highest increase in Tc in AB, whereas Tc in TP and PA remained unchanged. Compared to the control condition (non-cooling) the increase in Tc was smaller in TP and PA but greater in AB. Pritchett et al. (2010) failed to demonstrate any significant effect on Tc and Tsk as well as on ThS between cooling and non-cooling groups.

#### Ice Slurry

The work written by Trbovich (2019) was the only study that examined the effect of ice slurry ingestion. The author detected a significantly smaller increase in Tc that was attributed to the use of ice slurry during exercise (0.3°C ice slurry vs. 1.0°C non-cooling). The amount of ice slurry ingested was not reported.

#### Combined Methods

Griggs et al. (2017a) examined the impact of combining the use of an ice vest and water spray during exercise. The results showed that this strategy could significantly reduce the rise of Tc (1.3°C vs. 1.9°C in cooling and non-cooling) and Tsk, but no significant effect on ThS was found.

#### Cooling and Performance

Three studies investigated the effect of PRE- or DUR on performance. Griggs et al. (2017a) and Pritchett et al. (2010) found no significant differences in performance, whereas Webborn et al. (2010) demonstrated a significantly improved mean exercise duration when using PRE or DUR compared to a non-cooling protocol in participants with TP (time to exhaustion: 52.8 vs. 47.2 vs. 36.2 min, in DUR, PRE and non-cooling). Interestingly the peak power output was significantly lower in DUR (181 W) compared to PRE (195 W) and non-cooling (196 W).

### Heat Acclimation

Two studies (Table 7) examined the effect of heat acclimation on different physiological parameters in individuals with PA. While Castle et al. (2013) investigated the responses of a seven consecutive day heat acclimation protocol with 20 min arm-cranking exercise and 40 min rest in the heat (~33°C, ~65% relative humidity) per day, Trbovich et al. (2016) choose a seven consecutive day heat acclimation protocol (~35°C, ~40% relative humidity) with 30 min arm-cranking and 15 min rest. Participants were either trained target-shooting athletes with a SCI (Castle et al., 2013) or untrained volunteers with a PA or TP (Trbovich et al., 2016). Each study examined the effects on heart rate, Tc, RPE, ThS and plasma volume. Additionally Trbovich et al. (2016) measured Tsk values and Castle et al. (2013) the sweat responses.

**Table 7:** Acclimation.

**Study**	**Sample**	**Climate**	**Intervention/Exposure**	**Duration**	**Thermal measurement**	**Methodological characteristics**	**Outcome**
Castle et al. (2013)	5 PA, 2 F, 3 M (C5-T10)	33.4 ± 0.6°C 64.8 ± 3.7% rh	7 consecutive day heat acclimation protocol 20' arm cranking and 40' rest or shooting	20' exercise 40' rest or shooting	T_au_ Sweat rate ThS Plasma volume HR	nRnCT cohort study	Positive changes in T_au_, ThS, plasma volume No changes in sweat rate and resting HR
Trbovich et al. (2016)	5 TP (C5-C7) 5 PA (T7-L3)	35°C ~ 40% rh	7 consecutive day heat acclimation protocol 30' arm cranking 50% P_peak_, 30' passive recovery	15' rest 30' exercise in heat 30' rest in heat	T_au_ T_sk_ ThS HR Plasma volume	nRnCT cohort study	T_sk_ forehead ↓ after 7 days in PA T_sk_ calf ↑ after 7 days in TP ThS ↓ after 7 day in TP/PA No differences in T_au_. HR, blood lactate and plasma volume between day 1 and 7

*M, male; F, female; PA, paraplegia; TP, tetraplegia; C, cervical; T, thoracic; L, lumbar; °C, degree Celsius; rh, relative humidity; T_au_, aural temperature; T_au_, skin temperature; HR, heart rate ThS, thermal sensation; nRnCT, non-randomized non-controlled trial; P_peak_, peak power output; ↑ = higher; → = indifferent; ↓ = lower*.

Trbovich et al. (2016) found no significant changes after a seven consecutive day heat acclimation in all measured parameters. Castle et al. (2013) demonstrated a significant decline in Tc during heat session (day 1: 37.2°C, day 7: 36.7°C). Heart rate, ThS and a significant increase in plasma volume (1.5%), after seven consecutive days of heat acclimation was detected as well. The other measured parameters did not show any variances.

## Discussion

The purpose of the present work was to conduct a systematic review of studies investigating thermoregulatory responses in individuals with SCI during exercise in temperate or hot conditions or by application of cooling methods or heat acclimation protocols. In total, 32 studies were included to answer those questions (Figure 1).

### Thermoregulatory Responses in Temperate Conditions

Gagge et al. (1967) already stated that a small increase in the environmental temperature can lead to a reduced thermal comfort as well as an increase in Tc (i.e., environmental temperatures beside thermal neutrality of 28–30°C in AB, lead to a rapid increase in thermal discomfort and this is correlated with an increased sweating response). The magnitude of the increase depends on the individual thermoregulatory abilities. Six studies identified a significantly higher maximal Tc or a higher rate of increase of Tc in TP. These values were significantly higher in TP compared to AB (Fitzgerald et al., 1990; Price and Campbell, 1999b; Theisen et al., 2000, 2001; Griggs et al., 2015a, 2017b) (Table 4). There is only one article that did not detect any significant difference in Tc between AB and TP (Au et al., 2019), a finding that was presumably caused by a low exercise intensity (50% VO_2peak_ reserve). The difference in Tc between groups in studies comparing AB and PA are mostly insignificant (Price and Campbell, 1997; Pritchett et al., 2011, 2015; Veltmeijer et al., 2014; Iturricastillo et al., 2016). It has been suggested, that the heat balance in AB and PA is similar and achievable potentially by greater relative sweat rates in PA (Price, 2006) or heat gain and heat loss is equal in those body parts where a sweating capacity exist (Price and Campbell, 2003; Price, 2006). Pritchett et al. (2011, 2015) chose a different approach and speculated that an earlier rise in the Tsk in PA participants leads to an earlier sweating response, which might help to compensate the lower ability to sweat. The included studies in this review provides contrasting results. Whereas, Theisen et al. (2001); Pritchett et al. (2011) reported from the beginning of the exercise a decrease in Tsk in AB and a slightly increase for PA, Griggs et al. (2017b) found an increase in Tsk for AB and a unchanged Tsk for SCI in the first half of the exercise. Nevertheless, Vanbeaumont and Bullard (1965) found that the onset and the magnitude of sweating of AB was strongly influenced by Tsk. This is in line with the presented results.

The described results are consistent with the knowledge of thermoregulatory and thermal responses at rest, in which PA individuals demonstrated a similar heat tolerance as AB controls (Price and Trbovich, 2018) despite the fact of having only ~50% of the available surface area for cooling purposes. Additionally, another very interesting aspect was that the Tc remained elevated up to 30 min in TP during post exercise recovery (Price and Campbell, 1999b; Griggs et al., 2015a). This confirms the inability to dissipate the heat produced during exercise, which remains stored in humans' body during recovery.

Findings for Tsk are less straightforward. Several studies reported significantly higher values in TP compared to AB or PA (Fitzgerald et al., 1990; Griggs et al., 2015a) and PA showed higher values than AB (Theisen et al., 2000, 2001; Pritchett et al., 2011). Interestingly, PA showed a lower increase in thigh Tsk, but greater increase in calf Tsk compared to AB, whereas both calf and thigh Tsk were generally lower in SCI (Price and Campbell, 1997, 1999b). The disturbance in the redistribution of blood due to the lack of sympathetic vasoconstriction below the lesion level, and the inability to activate the muscle pump of the legs may be a reason for these findings (Hopman et al., 1992). Nevertheless, other studies failed to find differences between subject groups in Tsk of the lower limbs (Veltmeijer et al., 2014; Griggs et al., 2017b; Au et al., 2019). A comparison of these results presents some difficulties due to the different methods used in the studies. Tsk values were often reported as mean Tsk and not as regional values, thereby masking potential local differences between TP, PA and AB individuals (Price, 2006). Price and Campbell (1999a) speculated that convective cooling as a result of moving the arm relative to the body during propulsion could explain a decreased Tsk during wheelchair exercise (60% VO_2peak_), which is in line with the finding by Gass et al. (1988). Au et al. (2019) showed that the upper limb Tsk decreased in PA and TP participants during arm cranking as well. The authors suggested that the low fitness level (e.g., recreational) and thus the related low power output as well as the low intensity (e.g., 50% VO_2peak_ reserve ≜ 0.28 L^.^min^−1^, 0.55 L^.^min^−1^, 0.82 L^.^min^−1^, for TP, PA, or AB) were the reasons for the absence of exercise-induced heat stress in this study.

Fluid loss or sweat rate was monitored in several studies (Price and Campbell, 1997, 1999a,b; Griggs et al., 2015a, 2017b; Pritchett et al., 2015). To our knowledge, the work by Pritchett et al. (2015) was the first to analyze the difference of sweat gland density per cm^2^ and the associated sweat secretion rate between individuals with SCI and AB controls during exercise. Due to the smaller surface area available for sweating, a greater number of sweat glands or sweat output per gland could have been expected in individuals with SCI above the lesion level. However, participants with a SCI showed a lower sweat gland density per cm^2^ above the lesion levels compared to the AB controls. Intriguingly, the secretion rate of the single glands did not differ (Pritchett et al., 2015). Price and Campbell (1997, 1999a,b) detected a similar fluid loss between AB and PA individuals, whereas the only participant with TP had a lower fluid loss. Griggs et al. (2017b) could confirm the lower fluid loss in TP compared to non-SCI individuals. However, there is still some inconsistency, where AB and PA present a similar fluid loss, even though PA have a lower sweat glands density but the same secretion rate per glands as AB. Pritchett et al. (2015) hypothesized this equality may be a reason of a tendency toward a larger activity of a single sweat gland in SCI population. This would explain the similar fluid loss. Additionally, it has been noted that the density and capacity of sweat glands to produce sweat differs from one region to another (Sato and Sato, 1983). In the study by Pritchett et al. (2015) the simulated climate did not elicit a large thermal strain to trigger a sweat response on all measurement sites. In contrast it is quite clear that TP are sweating significantly less compared to AB and PA, which is in line with the higher lesion level and therefore in the loss of motor and sensory innervation to some regions of the arms and all regions of the legs, hips and trunk (Sato and Sato, 1983; Gass et al., 1992; Pritchett et al., 2015). Nevertheless, further studies are needed to investigate this topic in more detail.

### Thermoregulatory and Thermal Responses in Hot Conditions

None of the studies showed a difference in the increase of Tc between low lesion level PA and AB participants. These findings support the conclusion that individuals with a low level SCI are able to regulate their temperature in hot conditions similar to AB individuals (Hopman et al., 1993; Dawson et al., 1994; Boot et al., 2006; Forsyth et al., 2019). Petrofsky (1992) found a significantly higher Tc in SCI (PA and TP) participants during exercise at both 35.0° and 40.0° C with 50% relative humidity, compared to AB counterparts. The authors hypothesized this was mainly due to ineffective (e.g., no evaporation, sweat was running off the body) sweating.

Hopman et al. (1993) and Forsyth et al. (2019) both found a significantly higher rise in Tc in PA participants with a high lesion level (high lesion PA ΔTc ~ 1.2° C, low lesion PA ΔTc ~ 0.6° C, AB ΔTc ~ 0.3° C) compared to AB or PA participants with a low lesion level. As opposed to this, Boot et al. (2006) and Price and Campbell (2003) were unable to support these findings. The discrepancies between the studies can mainly be explained by differences in methodology (see Table 5) e.g., different relative workloads, different duration or different exercise type which may provide differences in heat production and therefore not enough heat gain and a related effect on Tc. Forsyth et al. (2019), Petrofsky (1992) and Price and Campbell (2003) included TP in their studies. The Tc of the TP participants increased significantly more than in AB or PA participants (ΔTc, TP 1.9–2.1°C, PA 0.9–1.3°C, AB 0.3–0.5°C). This emphasizes the statement previously made; individuals with a high lesion, notably individuals with TP, are not able to regulate their temperature during exercise in hot conditions. This is explained by the dramatically reduced surface area of the skin which is capable of secreting sweat, resulting in a larger heat storage as well as in a faster increase in Tc. Interestingly, the Tc remained elevated throughout 30 min of recovery from exercise (Price and Campbell, 2003).

Four studies have reported Tsk (Dawson et al., 1994; Price and Campbell, 2003; Boot et al., 2006; Forsyth et al., 2019). Dawson et al. (1994) observed a significant increase of the mean Tsk in PA (ΔTsk 3.6°C in PA, 2.3°C in AB) compared to AB individuals. While Tsk reached a steady state in AB, it rose continuously in PA. When differentiating between upper and lower body Tsk Boot et al. (2006) found a significantly larger increase in Tsk in the lower body when comparing PA and AB participants, while upper body Tsk was not significantly different. The activation of sweat glands is not possible due to their location below the lesion level and therefore, the absence of evaporation in this body region leads to a steady rise of Tsk. This is in accordance with the absence or reduction of sweat glands activity found by Pritchett et al. (2015). Finally, Price and Campbell (2003) could not detect any differences in forehead-, forearm-, upper-arm-, back-, chest-, abdomen-, calf- and thigh-Tsk between individuals with a high and low lesion PA. Due to the difference in lesion level, only in the abdomen a difference between the two groups could be expected. Therefore, the authors concluded that the absence of a difference may be caused by the design of the wheelchair (e.g., height of the backrest and the type of sport the wheelchair is used, which has to be taken in consideration) (Price and Campbell, 2003).

Forsyth et al. (2019) and Price and Campbell (2003) investigated Tsk not only in individuals with a high or low lesion PA, but also in TP. They observed a significant difference in the increase of local Tsk when comparing TP with AB participants (Forsyth et al., 2019) and TP with PA (Price and Campbell, 2003), respectively. Two different possibilities were present: Individuals with TP demonstrated greater changes in upper and lower body Tsk than AB controls (Forsyth et al., 2019), while Price and Campbell (2003) found a difference in the upper body parts (i.e., back-, chest-, forehead Tsk) between TP and PA. A possible reason may be the higher ambient temperature (35 vs. 31°C) in the study by Forsyth et al. (2019). Due to the warm blood from the active muscle perfusing the skin and the influence of ambient temperature in the upper body Tsk exceeded 35°C in all groups (Forsyth et al., 2019). In the lower body parts the temperature did not exceed 35°C. The authors suggested that the lower baseline Tsk and therefore the larger capacity in the lower body parts could be the reason for this difference. A lower active muscle mass could present a possible explanation and therefore the higher ambient temperature has a more pronounced impact on Tsk. A smaller increase in thigh Tsk in PA may be explained by the loss of vasomotor control (Price and Campbell, 1999b). In TP this is even more evident. In areas where insensate skin sweating does not occur, heat cannot be dissipated, which results in a continuous increase in Tsk (Price and Campbell, 2003).

Six of the included studies that analyzed the thermoregulatory and thermal responses in hot conditions, observed fluid loss or sweat rate (Petrofsky, 1992; Hopman et al., 1993; Dawson et al., 1994; Price and Campbell, 2003; Boot et al., 2006; Forsyth et al., 2019). Boot et al. (2006) detected a significantly lower fluid loss in high lesion level compared to low lesion level PA and AB controls, respectively. Moreover, the low lesion level PA had a significantly lower fluid loss compared with AB controls. Hopman et al. (1993) found a similar fluid loss as Boot et al. (2006), but interestingly, split up the individuals with PA into three groups according to their lesion level (high-PA: T1-T6; mid-PA: T7-T9; low-PA: T10-T12). Thereby, a significantly higher fluid loss in AB compared to all PA groups was evident, but the higher the lesion level, the lower the fluid loss was (fluid loss AB: 697 g, high-PA: 231 g, mid-PA: 351 g, low-PA: 439 g). Dawson et al. (1994) could not detect any significant differences in fluid loss between AB and PA. Dawson et al. (1994) argued that the clothing may have an influence. Price and Campbell (2003) showed that there is a greater fluid loss in PA compared to TP. They suggested that the greater fluid loss of PA would result from a greater body surface area available for sweating. Petrofsky (1992) showed that the mean body sweat rate was linearly related to the environmental temperature in each group (i.e., the higher the temperature the higher the fluid loss). Additionally, they detected a larger sweat rate in AB participants followed by PA and TP. The investigators (Petrofsky, 1992) could not find any sweat secretion below the lesion level. Consequently, the sweat must have been produced by the non-paralyzed body region. Additionally, they observed a six-fold higher regional sweat rate compared to AB. Nevertheless, most of the sweat ran off the body, making it ineffective for heat loss. In addition, it was the only study which reported such high sweat rates in TP participants and therefore drawing firm conclusions seems inappropriate. These findings stand in contrast to the similar sweat secretion per glands found by Pritchett et al. (2015). Forsyth et al. (2019) did not find any sweat secretion in individuals with TP on their forehead and upper back. There was a larger local sweat rate in high lesion level participants compared to low lesion level athletes and AB participants. Thus, the proposal by Petrofsky (1992) that there may be a compensatory larger increase in sweat rate above the lesion level could not be confirmed by those findings. The very contrasting results may be caused by the difference in exercise intensity. The heat produced during exercise depends directly on the exercise intensity (Gleeson, 1998), which was shown to have a large influence on sweat rate in AB (Baker et al., 2019). Additionally the responses found by Forsyth et al. (2019) can't be directly compared between TP and AB due to differences in the target heat production (i.e., 4.0 vs. 6.0 W/kg).

In summary, the present data demonstrates the graded effect of regional denervation among individuals with a SCI and substantiates that the greater increase in Tc in TP and PA with a high lesion level is evidentially the result of impaired sweating (Forsyth et al., 2019), which stands in line with the consent of other included literature (Petrofsky, 1992; Price and Campbell, 2003; Boot et al., 2006). But, there is still a lot of debate on the exact mechanism and future studies should further explain the responses during sport-specific conditions including protocols that mimics the duration of training or competitions.

### Cooling

#### Cooling/Ice Vest

The use of cooling garments, such as cooling vests, is becoming more and more common in the AB athletic population (Jones et al., 2012). Several studies (Uckert and Joch, 2007; Quod et al., 2008) indicated that the cooling/ice vests reduces Tsk, while Tc remains unaffected. In the SCI population Griggs et al. (2017a) observed in TP, the effect of using a cooling vest before (PRE) performing an intermittent sprint protocol and found no effect on Tc and performance. Webborn et al. (2005, 2010) reported that wearing an ice vest PRE and DUR significantly reduced the increase of Tc and ThS in TP. In addition, Webborn et al. (2010) showed that this cooling intervention improved performance on an intermittent arm crank sprint protocol in the heat. Participants were able to complete lager number of sprints and reached therfore a longer duration. The reason for this discrepancies in findings may be the difference in environmental conditions and exercise type. The study by Webborn et al. (2010) was performed in a lab with hot conditions on an arm crank ergometer and wheelchair tennis players, the study by Griggs et al. (2017a) in a sport hall and therefore more ecologically valid, with temperate temperatures and in wheelchair rugby chairs. Another reason for the longer exercise time Webborn et al. (2010) observed for DUR, could be the significantly lower power output in the initial sprints compared to PRE or non-cooling. The other studies which used a cooling vest as a DUR method did not report any significant differences in increase of Tc between groups in hot conditions (Armstrong et al., 1995; Bongers et al., 2016; Trbovich, 2019). These results are in line with the findings made in studies with Olympic athletes, where mainly the Tsk was lowered without any effect on Tc (Ross et al., 2013).

To date, only one study has examined the effect of wearing cooling vest only as a DUR device in a field setting (Trbovich et al., 2014). A cooling vest with phase changing material was used during 60 min of wheelchair rugby or basketball games in PA and TP. Even though Tc in individuals with TP increased to a higher extent compared to PA, cooling vests showed no effect on Tc in both groups. Compared to the PRE field setting Griggs et al. (2017a) used, TP reached similar Tc values and therefore participants have been thermally challenged to a similar extent in both studies. Whereas wearing an ice vest during intermitted sprints has beneficial effects, the use of this cooling technique during steady-state exercise, did not had beneficial effects on performance and Tc (Armstrong et al., 1995; Trbovich et al., 2014; Bongers et al., 2016).

Wearing a cooling vest DUR has lowered the ThS score in the studies by Bongers et al. (2016) and Webborn et al. (2005, 2010). In contrast, using an ice vest PRE did not influence ThS (Webborn et al., 2005, 2010). This might lead to the conclusion that using an ice vest DUR may give the athletes a positive feeling from a psychological perspective. Nonetheless, if Tc remains at the same level, it could be potentially dangerous for unnoticed overheating.

#### Other Cooling Garments and Devices

Armstrong et al. (1995) observed the effect of a refrigerated head piece which was placed on participants' (PA) head during 30 min of exercising at self-selected 10-km race pace intensity. Compared to the placebo condition or the use of an ice vest, a refrigerated head piece did not significantly influence Tc. The authors concluded that the efficiency of this head piece was not optimal regarding heat transfer. In AB it was shown that cooling the areas of the head and neck during cycling (Ansley et al., 2008; Schlader et al., 2011) or running (Tyler et al., 2010; Minniti et al., 2011) appeared to induce sizeable time-trial performance (up to 6%) or exercise capacity (up to 51%) improvements in the heat, despite not inducing significant changes in thermoregulatory and thermal responses. This could be explained through a greater density of cold-sensitive thermal afferents in the area of the head (Stevens et al., 2017). To investigate psychological effects of cooling strategies, it would be interesting to cool the sensate skin areas (e.g., head and neck) in athletes with TP.

Hagobian et al. (2004) was the only included article which used a cooling device on the feet during upper body exercise. Even though the rise in Tc was attenuated during exercise, it is unclear if individuals with an SCI may benefit from this kind of cooling device. Additionally, this method might not be practical in a field setting and it could be dangerous for local hypothermia or frostbites due to the insensitivity of the legs.

#### Water Spray and Artificial Sweat

Sprayed-on liquid transfers heat into vapor and has a similar effect as sweat evaporation (Trbovich et al., 2019). Therefore, using water spray as a cooling strategy may also slow the rise in Tc. The application of water spray (17°C) as artificial sweat during an arm cranking ramp protocol in participants with PA showed no effect on Tc, Tsk or ThS (Pritchett et al., 2010). Nevertheless, four participants slightly improved their time to exhaustion. Due to a larger thermal capacity it is suggested that a lower water temperature may have a greater effect, and might contribute to the lower ThS.

In a case study (Trbovich, 2019) water spray mitigated the rise of Tc in one TP and one PA and was, compared to the ice slurry and ice vest methods, the most effective strategy. Unfortunately, the temperature of the water used was not displayed and the suggestion that a lower temperature may have a greater effect cannot be verified. The effect of water spray with a larger number of participant was assessed by another study of Trbovich et al. (2019). They showed that the increase of Tc could be significantly decelerated in TP but not in PA. Spraying liquid on areas that already sweat was shown as beneficial as well, but the effect was smaller compared to those body areas with no sweating response (Trbovich et al., 2019). Further investigations are needed to verify the efficiency regarding thermoregulation and performance.

#### Ice Slurry

The use of an ice slurry in AB athletes is a well-established PRE and DUR method for lowering Tc and to enhance performance during endurance exercise (Jones et al., 2012; Stevens et al., 2017). In athletes with SCI only one case study has been performed. Trbovich (2019) tested the effect of ice slurry on thermoregulatory and thermal responses during activity in one participant with PA and one with TP and compared it to other cooling methods. The ice slurry lowered the increase in Tc in individuals with PA and TP in the hot condition. Jay and Morris (2018) found that ice slurry ingestion during exercise reduces sweat rate in AB. Therefore, the cooling benefits are likely greater for individuals with a lack in sweating response such as those with a SCI. As a consequence the use of ice slurry as a PRE or DUR could be an interesting strategy in SCI and needs some further investigations. It has to be noted, though, that especially persons with SCI have to test the gastrointestinal comfort and have to find the optimal dose in terms of volume ingested due to a slower gastrointestinal transit time.

#### Combination of Methods

Griggs et al. (2017a) tested the combination of two cooling methods. The use of an ice vest as PRE device and water spray during intermittent sprint performance was more effective to lower thermal strain compared to PRE with an ice vest only (ΔTc combined 1.3°C, only PRE 1.7°C, non-cooling 1.9°C). A limitation of the study was that the investigators did not study the effects of using water spray only during exercise. Also, in AB a combination of different methods has been shown to be an effective strategy to lower Tc and enhance performance (Jones et al., 2012) more effectively.

### Acclimation

Heat acclimation leads to several physiological adaptations (i.e., a lowered body Tc, enhanced skin blood flow, increased sweating capacity, reduced cardiovascular strain, augmented cellular protection, improved fluid balance, and altered metabolism) in AB individuals which improve thermoregulation, lower physiological strain, reduce the risk of heat-related injuries and improve performance in endurance exercise in hot environments (Tyler et al., 2016; Casadio et al., 2017; Racinais and Periard, 2020). The number of heat exposures, the environmental conditions, the duration, intensity and frequency of exercise, determine the magnitudes of these adaptations (Wendt et al., 2007; Garrett et al., 2012; Periard et al., 2015). Due to compromised sudomotor and vasomotor activity below the lesion level, it seems unclear if individuals with a SCI are able to acclimate or acclimatize to the same extent as AB. Castle et al. (2013) showed that during a seven consecutive day heat acclimation protocol with a heat exposure of 60 min including 20 min of low-intensity exercising, trained athletes with PA were partly able to heat acclimate. Heart rate, Tc, sweat rate and plasma volume were adapted following heat exposure. On the other hand, Trbovich et al. (2016) found no significant adaptation in untrained PA and TP during a seven consecutive day heat acclimation protocol with a heat exposure of 60 min including 30 min of low-intensity exercise. It has to be noted that in the study by Castle et al. (2013) the participants were trained and only individuals with PA were included. Individuals with PA typically have greater vaso- and sudomotor activities than TP. Trbovich et al. (2016) included only untrained subjects with PA and TP and it was argued that trained subjects may have undergone some prior acclimation experience. Furthermore, it is indicated that AB athletes need around 14 days to complete most adaptations (Periard et al., 2015). Therefore, a longer heat acclimation protocol is suggested with trained and untrained TP and PA to draw any further conclusions.

### Limitations of the Studies

All but three included studies were of moderate to strong quality (Table 3). In the context of the quality assessment, most studies lost scoring points due to a non-appropriate participant selection (i.e., missing inclusion/exclusions criteria, unclear sampling strategy, etc.) or due to a small sample size and an associated low power. The recruitment of an appropriate number of participants is very difficult since individuals with SCI represent a small group in the general population. A low power and therewith, the chance of detecting a true error as well as the chance that significant results reflect a true effect are decreased and therefore an overestimation of the effect sizes is common (Button et al., 2013). Therefore, we suggest to include multicenter studies to reach a representative statistical power in future research. Another limitation is the impossibility of blinding a participant to the chosen cooling strategy or the chosen environmental conditions. This may affect their behavior as well as heat sensations during the trial.

Overall the included studies were very heterogeneous, thus a comparison was limited by different methods to measure Tc, differences in cardiovascular fitness level of the participants and the high variation concerning chosen exercise type and duration as well as the environmental conditions. The level of cardiovascular fitness has a significant influence on thermoregulatory and thermal responses during exercise in thermal neutral conditions (Greenhaff, 1989) and additionally cardiovascular training leads to a decreased threshold for sweating (Gleeson, 1998) and therefore to a greater dissipation of the heat and consequently to a slower increase of the Tc. Another aspect is the short duration exercise (i.e., 16 min) (Iturricastillo et al., 2016) investigated. This could lead to a lower increase of Tc and therefore not inducing a thermal stress resulting in different outcomes between, TP, AB and especially low and high level PA. A major problem in the comparability of the groups is the confounding influence of individual differences in metabolic heat production and body size. The recent work by Forsyth et al. (2019) highlights an exercise method (i.e., fixed heat production), which allows a better comparison and shows a smaller variability in individual responses based on injury level. Furthermore a small difference in the environmental temperature can lead to a dissimilar thermal discomfort as well as large differences in thermoregulatory and thermal responses (Gagge et al., 1967). Therefore, different investigated environmental conditions makes any study comparison even more complex.

In summary, the exercise intensity levels used in the studies may therefore be below a certain threshold needed to generate enough heat to find differences between the groups. Thus, an exercise intensity below 60% of VO_2max_ might result in such a critical threshold in studies with SCI (Price, 2006). Therefore, future studies should investigate heat stress at an exercise intensity above 60% VO_2max_ and a duration of more than 25 min.

### Future Research

It is evident that the impairment of thermoregulatory functions is a consequence of the transection of the spinal cord. The consequences of a SCI on exercise and cardiovascular as well as on thermoregulatory function and thermal responses are well-studied. Still, there is a potential for further research to clarify these issues (e.g., the local Tsk, local sweat rate, sweat glands density, secretion rate per glands in PA, TP and the difference between those groups and AB). Additionally, differences concerning local sweat rate between AB, PA and TP and the perceptual aspects of thermoregulation remain unclear. Studies should employ practical settings and protocols which mimic the real-life situation as close as possible. The very new methodological way (i.e., exercising with a fixed heat production) chosen by Forsyth et al. (2019) needs to be highlighted. Due to de fixed heat production, the quantification of influence of SCI level on changes in thermoregulatory and thermal responses during exercise independent of biophysical factors, would be possible in a better way. Therefore, further research using a fixed heat production as exercise intensity is recommended.

In AB persons the field of cooling strategies seems well-investigated. Cold water immersion, ice slurry and the combination of different methods are shown to be the most effective strategies for lowering Tc and enhancing performance. In SCI, ice slurry has only been tested in a limited setting and regarding cold water immersion, there is no existing literature at this point (Bongers et al., 2017).

Whether individuals with SCI, especially individuals with TP, can acclimate still remains unclear. Longer heat acclimation protocols with at least 14 days, trained and untrained, TP and PA should be performed to be able to recommend heat acclimation in SCI or not.

The authors would like to draw attention to implement more studies with female participants. The menstrual cycle may play an important role in any thermoregulatory and thermal responses (Giersch et al., 2020).

In general, more than half of the included literature is older than 15 years and the methods of some investigations are no longer “gold standard” (e.g., measurements of Tc, Tsk, sweat rate, etc.). This should not discount those findings, but it shows that future research is warranted.

### Summary of Main Findings

The current systematic review has investigated the thermoregulatory and thermal responses of individuals with a SCI during exercise in different conditions. Furthermore, the use of PRE and DUR cooling methods and heat acclimation and its effects on thermoregulatory and thermal responses have been studied intensively. A total of 32 articles have been identified. The reviewed literature showed no thermoregulatory and thermal disadvantages in PA (high and low lesion level PA) compared with AB during exercise in temperate conditions. For both populations the initial increase in Tc is followed by a plateau. The dynamics of the Tsk is similar in AB individuals, but PA persons with a high lesion level showed a larger increase in Tsk, due to greater amount of heat storage and a lower sweat response. On the other hand, it seems evident that TP individuals have a continuous increase in Tc with no plateau during exercise in temperate conditions. No difference in Tsk between PA with a high lesion level and TP were found. Individuals with PA showed a lower sweat rate than AB but a higher one compared with persons with TP. The reasons for this might include the smaller sensitive skin area and the lower sweat gland density compared to AB.

During exercise in a hot environment the increase in Tc is dependent on the conditions and the exercise intensity and does not differ between AB and low-level PA. Possibly in high-level PA and certainly in TP the Tc increases to be faster and to a higher extent compared to AB and low-level PA.

The return to baseline Tc in SCI is slower in hot conditions. In TP and high-level PA the Tc remains above the level observed for other groups and needs more time to return to the baseline level. In the insensate skin areas Tsk increased continuously due to the lack of sweating. The overall sweat capacity decreases the higher the lesion level is. Individuals with a TP have only a small or even no sweat secretion at all.

To reduce thermal strain two methods are used: Firstly, the application of cooling methods PRE and DUR an activity, secondly, heat-acclimation. Due to compromised vaso- and sudomotor activity below the lesion level, it is not clear if PA and TP are able to acclimate to heat to the same extent as AB. Therefore, it seems difficult to give any concrete recommendation for optimal acclimation strategy for individuals with a SCI, predominantly due to the lack of studies in this field. The use of cooling techniques showed a tendency to be useful in reducing ThS even if Tc is not substantially reduced. Nevertheless, a recommendation for an optimal strategy is premature. It should be noted that for each athlete with SCI, the consequences of the injury should be investigated individually and personal recommendations should be tailored based on these physiological consequences for each athlete (Griggs et al., 2015b).

### Conclusion

– Individuals with a high-level SCI, especially those with a TP, had larger increases in Tc and Tsk values.
– Tsk data show the graded effect of regional denervation and reinforces that the greater increase in Tc is the result of the impaired sudomotor function.
– The use of cooling techniques before and during exercise can positively affect the burden of the impaired thermoregulatory system of individuals with a TP or PA.
– Implementation of acclimation protocols should be investigated in the future before any recommendations can be given.
– Individuals with TP require special attention during exercise in temperate and especially in hot conditions to prevent them from potential heat related injuries due to potentially higher values in Tc and Tsk.

### Practical Applications

Temperate conditions (15–25°C):

– Monitor heat strain especially in athletes with TP (e.g., telemetric pill to monitor the Tc, tympanic thermometers)
– Use the combination of cooling vests before and water sprays during exercise (i.e., team sports)

Hot conditions (>25°C):

– Monitor heat strain in PA with a high lesion level and athletes with TP (e.g., telemetric pills to monitor the Tc)
– Use of cooling methods to delay the increase in Tc
– Use of cooling vests, ice slurry before and during activities if tested before the competition
– Test the use of cooling head piece and decide individually if its lead to an improved thermal comfort

Future research needs to take the following recommendations into account:

– Focus on sports specific, field based setting
– Use new methodological protocols (i.e., fixed heat production) for better comparability between groups with different lesion levels [as proposed by Forsyth et al. (2019)]
– Chose protocols with a sufficient duration or intensity
– Chose environmental conditions, which are comparable to other studies
– Need of multicenter studies to increase number of participants and power, meaningfulness of the results and a higher overall quality.

## Data Availability Statement

The original contributions presented in the study are included in the article/supplementary material, further inquiries can be directed to the corresponding author/s.

## Author Contributions

FG, JF, CP, BR, and RM: conception and idea. FG: literature research. FG, JF, and CP: literature rating. FG: draft manuscript preparation. BR, JF, and CP: supervising. All authors reviewed the results and approved the final version of the manuscript.

## Conflict of Interest

The authors declare that the research was conducted in the absence of any commercial or financial relationships that could be construed as a potential conflict of interest.
